# Enhanced Expectation of External Sensations of the Chest Regulates the Emotional Perception of Fearful Faces

**DOI:** 10.3390/brainsci11070946

**Published:** 2021-07-19

**Authors:** Won-Mo Jung, In-Seon Lee, Ye-Seul Lee, Yeonhee Ryu, Hi-Joon Park, Younbyoung Chae

**Affiliations:** 1 Acupuncture & Meridian Science Research Center, Kyung Hee University, Seoul 02447, Korea; jungcro@gmail.com (W.-M.J.); inseon.lee@khu.ac.kr (I.-S.L.); acufind@khu.ac.kr (H.-J.P.); 2 Jaseng Spine and Joint Research Institute, Jaseng Medical Foundation, Seoul 06110, Korea; yeseul.j.lee@gmail.com; 3 KM Fundamental Research Division, Korea Institute of Oriental Medicine, Daejeon 34054, Korea; yhryu@kiom.re.kr

**Keywords:** acupuncture, attention, fear, fMRI, default mode network, predictive coding

## Abstract

Emotional perception can be shaped by inferences about bodily states. Here, we investigated whether exteroceptive inferences about bodily sensations in the chest area influence the perception of fearful faces. Twenty-two participants received pseudo-electrical acupuncture stimulation at three different acupoints: CV17 (chest), CV23 (chin), and PC6 (left forearm). All stimuli were delivered with corresponding visual cues, and the control condition included visual cues that did not match the stimulated body sites. After the stimulation, the participants were shown images with one of five morphed facial expressions, ranging from 100% fear to 100% disgust, and asked to classify them as fearful or disgusted. Brain activity was measured using functional magnetic resonance imaging during the facial expression classification task. When the participants expected that they would receive stimulation of the chest (CV17), the ratio of fearful to non-fearful classifications decreased compared to the control condition, and brain activities within the periaqueductal gray and the default mode network decreased when they viewed fearful faces. Our findings suggest that bodily sensations around the chest, but not the other tested body parts, were selectively associated with fear perception and that altering external inferences inhibited the perception of fearful faces.

## 1. Introduction

Interoceptive signals are transmitted through various visceral substrates, including those in the cardiac, gustatory, and inflammatory systems [1,2,3,4,5,6,7,8]. Interoception, which refers to the processing of internal signals reflecting the state of the body, is involved in affective processing. Specifically, emotions can be shaped by internal bodily states as communicated via interoceptive signals [9,10,11]. For example, the sensitivity of cardiac interoception, measured according to the accuracy of performance in the heartbeat detection task, has been related not only to the intensity of emotional feelings [12,13,14] but also to pathological conditions such as borderline personality disorder [15]. Recently, the associations between cardiac signals and fear/threat processing have gained increasing attention from researchers. Previous reports have demonstrated that the phasic signal of the arterial baroreceptor selectively enhances the emotional impact of fearful stimuli [1,16]. Further, participants rated fearful facial expressions as significantly more intense when the faces were presented at the systolic versus diastolic phase, indicating that cardiac timing modulates the subjective perception of fear [17]. In addition to visceral signals unconsciously transmitted from the brainstem [18,19], consciously accessible feelings associated with integrated visceral states can contribute to emotional processing [20,21]. For instance, investigations of somatotopic patterns of bodily sensations elicited by emotional stimuli indicated that sensations felt around the heart region were consistently associated with fear [22,23,24]. The spatial distribution of bodily sensation reflected the specific physiological state of each emotion [24]. Using a bodily sensation map, we found that somatotopic patterns of bodily sensation were associated with the degree of interoceptive accuracy [23]. Our previous study showed that perceptual processing of fearful faces was facilitated by enhanced interoceptive sensations elicited by a bodily imagery task associated with fear [25].

Current evidence suggests that cardiac interoception is delivered via two major pathways [18,26]. One is the transmission of baroreceptor signals through the vagal nerve, and the other is skin afferent projections that deliver indirect movement around the heart. Although these pathways are known to independently deliver cardiac interoception [26], inconsistent signals between the two pathways may diminish the brain’s response to cardiac signals [18]. Many recent studies have investigated the antagonistic link between exteroceptive and interoceptive awareness of the body [27,28,29,30]. As a result, it has been proposed that the interactions between interoceptive and exteroceptive signals play a role in emotional responses [31]. Additionally, when the cause of a sensory expectation is changed from an internal state to external stimuli, the facilitation of fearful face perception is transformed to inhibition. The concept of “predictive coding” is useful in the context of interoception as a way to generalize the influence of bodily sensations on affective perception [31,32]. Such inferences can determine the responses that best fit the predictive models developed to understand the hidden causes of sensory signals [33,34]. Our previous study demonstrated that pseudo-electrical acupuncture stimulation efficiently induced perceived sensation around the desired body area in the absence of actual stimulation and implicated the salience network in a predictive role, monitoring internal and external bodily states during inferences regarding somatosensation [35]. In the present study, we induce prior expectations regarding external bodily sensations in the participant group, and the bodily sensations can be caused by an external stimulus (i.e., pseudo-electrical acupuncture stimulation). Based on these findings, we expect that there might exist an inhibition relationship between exteroceptive and interoceptive awareness of the body.

The aims of the present study were to investigate whether expectation regarding exteroceptive somatosensation during pseudo-electrical acupuncture stimulation could influence emotional processing and to determine the brain regions involved in this process using functional magnetic resonance imaging (fMRI). We hypothesized that exteroceptive inferences regarding bodily sensations at a peripheral area around the heart could inhibit interoceptive fear processing, while the exteroceptive context of other regions might limit this effect. More specifically, we expected that pseudo-electrical acupuncture stimulation could inhibit perceptual processing of fearful faces since the participants expected the somatosensation to be applied to the chest, which disturbs cardiac interoception.

## 2. Methods

### 2.1. Participants

We used advertisements targeted at students of Kyung Hee University and Korea University to recruit a total of 22 healthy volunteers (8 women, age = 23.7 ± 3.3). None of the participants had a history of neurological, psychiatric, or other major medical problems, and no participants used medications at the time of the study. The participants were instructed not to drink alcohol or caffeine and not to take any medications on the day before the study. All participants received a detailed explanation of the experimental procedure and provided written informed consent. This experiment was conducted in accordance with the Declaration of Helsinki, and the protocol was approved by the Institutional Review Board of Korea University (KU-IRB-15-108-A-1).

### 2.2. Pseudo-Electrical Acupuncture Stimulation

We used pseudo-electrical acupuncture stimulation to induce the expectation of somatosensation. We conducted figurative visualization to imply the delivery of electrical stimulation, although no actual stimulation was applied (pseudo-stimulation). The participants were encouraged to believe that they were receiving a somatosensory stimulation. They were given the following information: “This study is assessing the newly developed Transdermal Micro-electric Current Stimulation Device, which is an advanced form of electro-acupuncture stimulation”. The participants were also told that they would receive a very weak electric current through the attached electrodes, and that the delivery of the stimulation would be accompanied by a visual cue [35].

We used acupoints on three different body parts, CV17 (chest), CV23 (chin), and the left PC6 (forearm), for pseudo-electrical acupuncture stimulation. According to the bodily sensation map, CV17 is located in the body region that is most strongly related to fear, while CV23 is located in the body region that is most strongly related to disgust [23]. CV17 acupoint, located at the middle of the chest, has been well-known to relieve psychological problems and autonomic dysfunctions [36,37]. Although stimulation at the other acupoint, PC6, is prescribed for relief of emotional stress [38,39,40], this site is not located on the emotion-specific bodily sensation map [23]. The study was conducted during fMRI. Before they entered the fMRI scanner, we attached sticker-type electrodes designed with a carbon-filled plastic snap (ClearScan; Vermed, Inc., Rockingham, VT, USA) to three different acupoints on the participants, CV17, CV23, and the left PC6, without any invasive processes. The order of stimulations was counterbalanced.

The participant viewed the visual cues on a screen reflected on a mirror located inside the scanner. The screen showed a dot flickering on the stimulated body site on a human body template. In the control condition, the flickering dot was shown outside of the body template. The flickering dot on the human body template informed the participant regarding the timing of the pseudo-stimulation. The detailed pseudo-stimulation procedures were described in our previous study [35].

We performed the two studies on the same day. In session 1, we investigated the psychophysical and psychophysiological responses to the cognitive component of electrical stimulation using fMRI. Results showed that expected electrical stimulation on the same areas without actual stimulation produced greater deqi sensation compared to the control condition, and cognitive components of cutaneous electrical stimulation resulted in greater brain activations in the salience networks, including the insula and pre-supplementary motor area [35]. Deqi sensation is one of the therapeutic sensations during mind–body interventions, including acupuncture [41]. The main manifestation of deqi sensation includes a combination of various sensations, including heaviness, numbness, soreness, distension, and spreading sensations, separating from the acute pain at the site of the needling [42]. In session 2, we conducted this study, investigated whether enhanced expectation of external somatosensation on the chest could influence emotional processing, and determined the brain regions involved in this process. In session 1, all pseudo-electrical stimulation without actual stimulation produced greater deqi sensation compared to the control condition (CV17: 11.8 ± 3.5, CV23: 9.7 ± 3.5, PC6: 8.2 ± 2.4, control condition: 3.1 ± 1.2) [35]. Since we found the distinct psychophysical and brain responses to pseudo-electrical stimulation in session 1, we strongly believe that the subjects were successfully made to believe that stimulation would be delivered. 

### 2.3. Emotional Face Stimuli and Emotion Judgment Task

**Figure 1:**
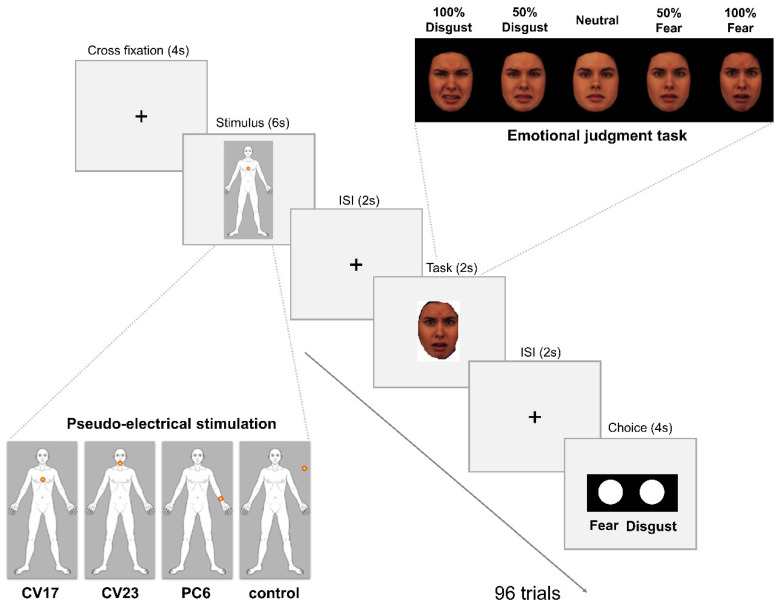
Experimental procedure during fMRI. Inside the fMRI scanner, pseudo-electrical acupuncture stimulation was delivered after visual fixation of a cross on a screen, and then the participants completed an emotion judgment task. The participants were informed that electrical acupuncture stimulation would be applied to three body parts, CV17 (chest), CV23 (chin), and PC6 (left forearm), and that no stimulation would be delivered in the control condition. All stimuli were delivered with corresponding visual information. Specifically, a dot was shown flashing on the screen on a model of the stimulation sites on the human body. In the control condition, the dot was shown flashing outside of the body model. After pseudo-electrical acupuncture stimulation, a face with one of five emotional facial expressions (100% fear, 50% fear, neutral, 50% disgust, 100% disgust) appeared for 2 s. A labeled button box was then shown on the screen, which corresponded with a real button box, and the participants were asked to complete an emotion judgment task. In this two-alternative forced-choice task, the participants were given 4 s to press a button to indicate whether they felt the facial expression was fearful or disgusted.

After pseudo-electrical acupuncture stimulation, the participants conducted an emotion judgment task while still inside the fMRI scanner. There were eight blocks with twelve trials each, comprising two blocks for each condition (stimulation on CV17, CV23, PC6, and the control site). Brain scanning took place after the participants had completed the first four blocks. In a two-alternative forced-choice task, the participants were instructed to classify emotional faces as either fearful or disgusted. The methods were described in part in our previous study [25]. The pseudo-electrical acupuncture stimulation was presented for six seconds after visual fixation on a cross on the center of the screen. After pseudo-electrical acupuncture stimulation, a fixation cross was displayed during an inter-stimulus interval ranging from 800 to 3200 ms (average 2000 ms). This was followed by a 2 s presentation of a face image, which was pseudo-randomly selected from five emotional facial expression stimuli (100% fear, 50% fear, neutral, 50% disgust, and 100% disgust). The images of emotional facial expressions were made from 24 pictures of eight individuals: four each of men with fearful, neutral, and disgusted faces and four each of women with fearful, neutral, and disgusted faces from the Karolinska Directed Emotional Faces database (http://www.emotionlab.se/resources/kdef (accessed on 3 June 2015)). Following a second inter-stimulus interval showing the fixation cross, participants were asked to classify the presented face as fearful or disgusted within 4 s (Figure 1).

### 2.4. Behavioral Analysis

The recognition ratio to fearful face during emotion judgment task was compared among four different conditions. The value was extracted from the differences in the recognition ratio to fearful face between the three pseudo-electrical stimulation conditions and the control condition. The statistical tests for the behavioral data were conducted using R 3.4.0 (http://cran.r-project.org (accessed on 13 May 2017)). The significance level was set to *p* < 0.05 and the *p* values were corrected with false discover rate.

### 2.5. fMRI Acquisition and Analysis

fMRI scans were completed using a 3-Tesla scanner (Siemens, Erlangen, Germany) with a three-axis gradient head coil. Blood oxygen level-dependent fMRI of the whole brain was obtained using a T2*-weighted gradient echo planar imaging (EPI) sequence with acquisition of 37 axial slices (TR = 2000 ms, TE = 30 ms, flip angle = 90°, field of view = 240 × 240 mm^2^, voxel size = 3.8 × 3.8 × 4.0 mm^3^). As an anatomical reference, a T1-weighted rapid gradient echo image dataset was generated using the following parameters: TR = 2000 ms, TE = 2.37 ms, flip angle = 9°, field of view = 240 × 240 mm^2^, voxel size = 0.9 × 0.9 × 1.0 mm^3^, and 192 slices. Stimulus presentation and response logging were performed using the PsychToolbox program in Matlab (MathWorks, Natick, MA, USA).

Preprocessing of the fMRI data was conducted using the Analysis of Functional NeuroImages (AFNI) software package [43]. The EPI data were corrected for slice time differences, realigned for motion correction, concatenated and transformed to a common space using the Talairach template [44], registered to the volume with the minimum outlier fraction, smoothed using a 6-mm full-width-at-half-maximum (FWHM) Gaussian filter, resampled to 3-mm isotropic resolution, and scaled to have mean of 100 for each voxel. The model was estimated by applying individual movement regressors.

We used a multiple linear regression with a gamma variate hemodynamic response function to analyze the fMRI data at the individual subject level. The regressors of interest modeled the fMRI signal response to the faces in the four conditions. The regressors were fitted to the scan time courses using the AFNI program 3dDeconvolve [36]. We examined the differences in the neural responses to the emotional faces among the different conditions. Contrast images were generated for each participant. To identify the brain responses elicited by fearful faces when expecting somatosensory stimulation at a fear-related region (CV17), we carried out a group-level analysis of brain responses observed during the pseudo-stimulation of CV17 (CV17 condition) versus that in the control condition. Similarly, brain responses elicited by fearful faces in other two conditions (CV23 and PC6) were compared to those in control condition. Cluster threshold corrections were applied using Monte Carlo simulations, and the results were evaluated using a family-wise error (FWE)-corrected significance threshold of p < 0.05 [45]. We used a modified version of the 3dFWHMx software with an auto-correlation function to extract the actual smoothness of the data for each participant. The 3dClustSim program was used with an uncorrected threshold of p < 0.005 for the volume correction simulations.

## 3. Results

### 3.1. Behavioral Responses to the Emotion Judgment Task

**Figure 2:**
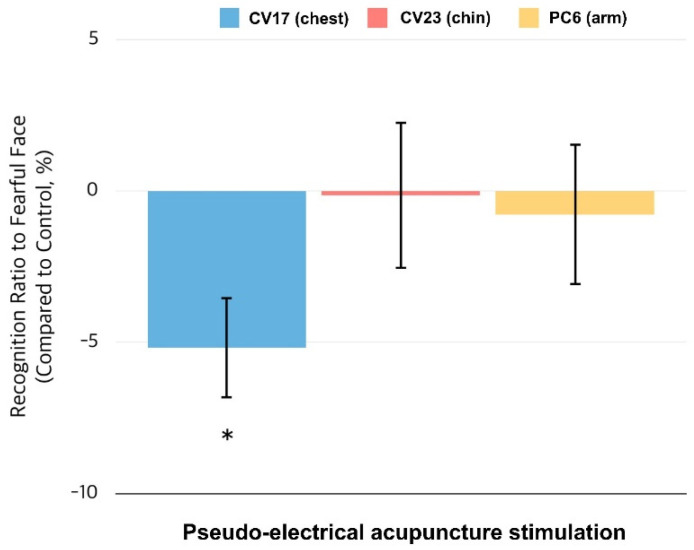
Proportion of faces classified as fearful compared with the control condition. We compared the proportion of faces classified as fearful in the three experimental conditions with that in the control condition. Only the CV17 (chest) condition elicited a significantly smaller ratio of fearful face classifications compared to the control condition. * *p* < 0.05.

We contrasted the group-level findings from the emotion versus control condition, as shown in Figure 2. The control-contrasted average of the classification ratio of fearful faces was −5.2 ± 1.9% for CV17, −0.15 ± 2.7% for CV23, and −0.78 ± 2.6% for PC6. A paired *t*-test revealed a significantly lower classification ratio for fearful faces in the CV17 condition only (*t* = 3.104, *p* = 0.018; false discovery rate (FDR) corrected), which indicates that the percentage of facial expressions perceived by the participants as fearful was lower in the CV17 condition than in the control condition. This effect was not found under the other stimulation conditions.

### 3.2. Brain Responses to Fearful Faces When Expecting Somatosensory Stimulation at CV17

**Figure 3:**
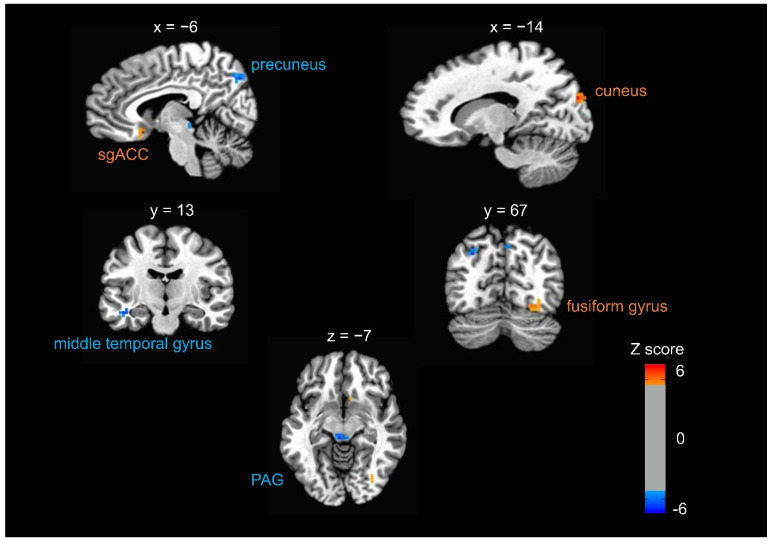
Brain responses to fearful faces in the CV17 versus control condition. The contrast between the CV17 condition and the control condition revealed that the brain activity elicited by viewing fearful faces changed under the condition of expecting somatosensory stimulation of the chest. When the participants viewed fearful faces in the CV17 condition, we observed significantly increased brain activity in the right cuneus, fusiform gyrus, and subgenual anterior cingulate cortex (sgACC), and significantly decreased brain activity in the bilateral periaqueductal gray (PAG) and precuneus and left middle temporal gyrus (MTG) (*p* < 0.05, FWE corrected) compared to the control condition.

Compared to the control condition, fearful faces elicited significantly decreased brain activity in the CV17 condition in the bilateral periaqueductal grey (PAG) and precuneus and middle temporal gyrus (MTG) (*p* < 0.05, FWE corrected). Compared to the control condition, fearful faces elicited significantly increased brain activity in the CV17 condition in the right cuneus, fusiform gyrus, and subgenual anterior cingulate cortex (sgACC) (*p* < 0.05, FWE corrected) (Table 1 and Figure 3). When we compared the brain responses to fearful faces in the other two conditions (CV23 and PC6) to the control condition, there were no significant results.

## 4. Discussion

Our data indicate that neural activity and behavioral emotional perception of faces, especially fearful faces, are influenced by the expectation of somatosensory stimulation of the chest but not other body parts. Participants were less likely to classify an emotional face as fearful after pseudo-electrical acupuncture stimulation on CV17 (chest) compared to stimulation at a control site. However, pseudo-stimulation of other body areas (chin and left forearm) did not lead to a behavioral change. Brain activity elicited by fearful faces, notably in the PAG and the default mode network, decreased in the CV17 condition compared to the control condition. We observed increased activity in response to fearful faces in the sgACC, cuneus, and fusiform gyrus.

These findings provide novel insight into the bidirectional action of interoceptive and exteroceptive body awareness on emotional processing. Particularly, our data indicate that fear perception was inhibited when participants were expecting a somatosensory stimulation (external sham stimulus) applied to the chest. However, this observation is in contrast with the previous finding that interoceptive inference around the chest area facilitated fear perception. In the previous study, interoceptive inferences regarding congruent bodily sensation patterns (an emphasis on sensations around the chest) enhanced the behavioral recognition of fearful faces and neural responses elicited by fearful faces [25]. This involvement of bodily sensations around the chest in the perception of fearful faces supported previous studies on the pivotal role of cardiac interoception in fear processing [1,16]. Specifically, the phasic signal of arterial baroreceptors at each cardiac systole facilitated the detection of fearful faces and enhanced the perceived emotional intensity of such faces [1]. Tsakiris and colleagues [30] tested the plasticity of body awareness using the rubber hand illusion, in which participants were encouraged to experience an artificial hand as their own via visual and tactile stimuli synchronously applied to the artificial and real hands. They reported that stronger body awareness for the fake hand was formed by weaker interoceptive sensitivity, suggesting that exteroceptive representation of the body may dominate interoceptive representation [30]. This negative correlation was also observed in a developmental study [28] and in a study with different manipulations of interoceptive processing [27,29]. This antagonistic relationship is intensified in individuals with pathological conditions that enhance their vulnerability to illusive somatosensation. For instance, a recent study investigated patients who experienced idiopathic skin sensations of insect infestation and found that these patients had poor sensitivity to a heartbeat signal and that they were highly sensitive to the rubber hand illusion [46]. These findings are consistent with the bidirectional relationship between interoceptive awareness of emotions and exteroceptive somatosensory signals during perceptual processing of fearful faces.

In our study, we found increased brain activities in sgACC, cuneus, and fusiform gyrus and decreased brain activities in the PAG and middle temporal cortex constituting the default mode network. It has been well known that the amygdala is the key brain region in the processing of emotional facial expression [25]. However, we were not able to find the change of brain activities in the amygdala in the current study. It is assumed that the emotional modulation by exteroceptive somatosensation induced by pseudo-electrical acupuncture stimulation might be more associated with a large scale allostatic–interoceptive system, default mode network, and brainstem visceromotor system rather than direct modulation of the limbic system. Our behavioral results are consistent with the neural responses observed for fearful faces under the CV17 (chest) condition compared with the control, as illustrated by the decreased activity in the PAG. The PAG is involved in regulating the autonomic substrates that allow behavioral adaptations, such as freezing and fleeing, which are associated with fear processing [47,48,49]. The PAG, a midbrain region involved in defense and emotional coping responses, is an important center for emotion-related autonomic and motoric processes [50]. The role of the PAG has been investigated in hyper-arousal and active defensive responses in the presence of fearful stimuli in individuals with post-traumatic stress disorder (PTSD) [51]. PTSD patients showed widespread PAG connectivity with regions involved in threat responses and emotional reactivity [52]. Interoceptive interventions have been used to improve a broad range of clinical disorders, including mental disorders [53]. Meditation practices can attend to sensations of the body through focusing on the breath by engagement of brain networks related with cognitive control and interoception and by disengagement from self-related thoughts [54,55]. Interoceptive awareness training, such as mindfulness-based intervention by cultivating attention to bodily sensations, reduced brain activations in the default mode network associated with mind wandering and self-referential processing [54,55]. Future research should explore distinctions between the neural substrates of interoceptive and exteroceptive bodily attention processing. Furthermore, we found increased neural responses in other regions, such as the sgACC, cuneus, and fusiform gyrus. These findings suggest that a large-scale allostatic-interoceptive system comprising the salience network, default mode network, and brainstem visceromotor regions, such as the PAG, could contribute to the modulation of emotional processing, depending on intrinsic and extrinsic bodily states [56].

Our study had some limitations. First, the neural mechanisms by which inferences regarding somatosensory stimulation of the chest area inhibit fear perception have yet to be comprehensively established. Thus, further investigation is required to examine the involvement of the salience network in somatosensory inferences that influence the part of the default mode network implicated in emotional processing. When we presented fearful and disgusted faces as emotional stimuli, fear was the emotion for which emotional recognition and the associated brain responses were reliably affected by somatosensory expectation of the chest. Thus, the selective association between the perception of fear and the body area around the chest should be investigated. Second, our study is an exploratory study on how enhanced bodily sensation of the chest regulates the emotional perception of fearful faces. Manipulations of brain functions, such as transcranial magnetic stimulation, will be necessary to confirm the brain mechanisms of expectation regarding exteroceptive somatosensation on emotional processing. Third, future studies are needed to investigate the associations between various categories of emotional stimuli and somatosensation induced in additional candidate body areas. Fourth, the number of participants in the current study was too limited to be interpreted in the general population. Since the low power of fMRI studies with a small sample size can exhibit exaggerated results, a larger sample size is necessary to ensure the reproducibility of this study. Finally, we did not further consider gender differences in our analysis due to the limited number of subjects. However, numerous studies have shown gender differences in cognitive and affective processing [57,58]. Thus, it will be interesting to further investigate the gender differences in emotion modulation in a future study with a larger sample size.

## 5. Conclusions

To the best of our knowledge, this study is the first to demonstrate that expectations regarding somatosensation induced by external sham stimuli of the chest area can inhibit fear perception. Our findings suggest that bodily sensations around the chest, but not other body parts, are selectively associated with fear perception and that altering external inferences can inhibit the perception of fearful faces. Moreover, brain activities in the PAG and the default mode network decreased when the participants viewed fearful faces under the condition of expecting somatosensory stimulation of the chest. Future research on manipulations of bodily sensations may integrate interventions for ameliorating pathological emotional processing.

## Figures and Tables

**Table 1:** Brain response to fearful faces when expecting somatosensory stimulation of the chest (CV17) compared to the control condition.

Activation	Location		Z Score	Voxels	Coordinates of Peak Voxel in Talairach Space (RAI)		
					X	Y	Z
Decreased activation	Precuneus	Left	−5.10	26	28.5	67.5	35.5
	Periaqueductal grey	Right /Left	−5.09	20	4.5	28.5	−6.5
	Precuneus	Right	−4.93	17	−4.5	73.5	38.5
	Precuneus	Left	−5.72	16	1.5	61.5	41.5
	Middle temporal gyrus	Left	−5.16	14	40.5	13.5	−15.5
Increased activation	Cuneus	Right	5.57	18	−13.5	85.5	23.5
	Fusiform gyrus	Right	4.49	16	−25.5	67.5	−12.5
	Subgenual anterior cingulate cortex	Right	4.5	10	−4.5	−13.5	−9.5

## Data Availability

Data are available on request from the authors.
